# Supplementary material for: HIV-1 adapts to lost IP6 coordination through second-site mutations that restore conical capsid assembly
Source: Nat Commun. 2024 Sep 13;15:8017. doi: 10.1038/s41467-024-51971-w (PMC11399258; doi:10.1038/s41467-024-51971-w)
Supplement: Supplementary file 1 — Supplementary Information [file 41467_2024_51971_MOESM1_ESM.pdf]

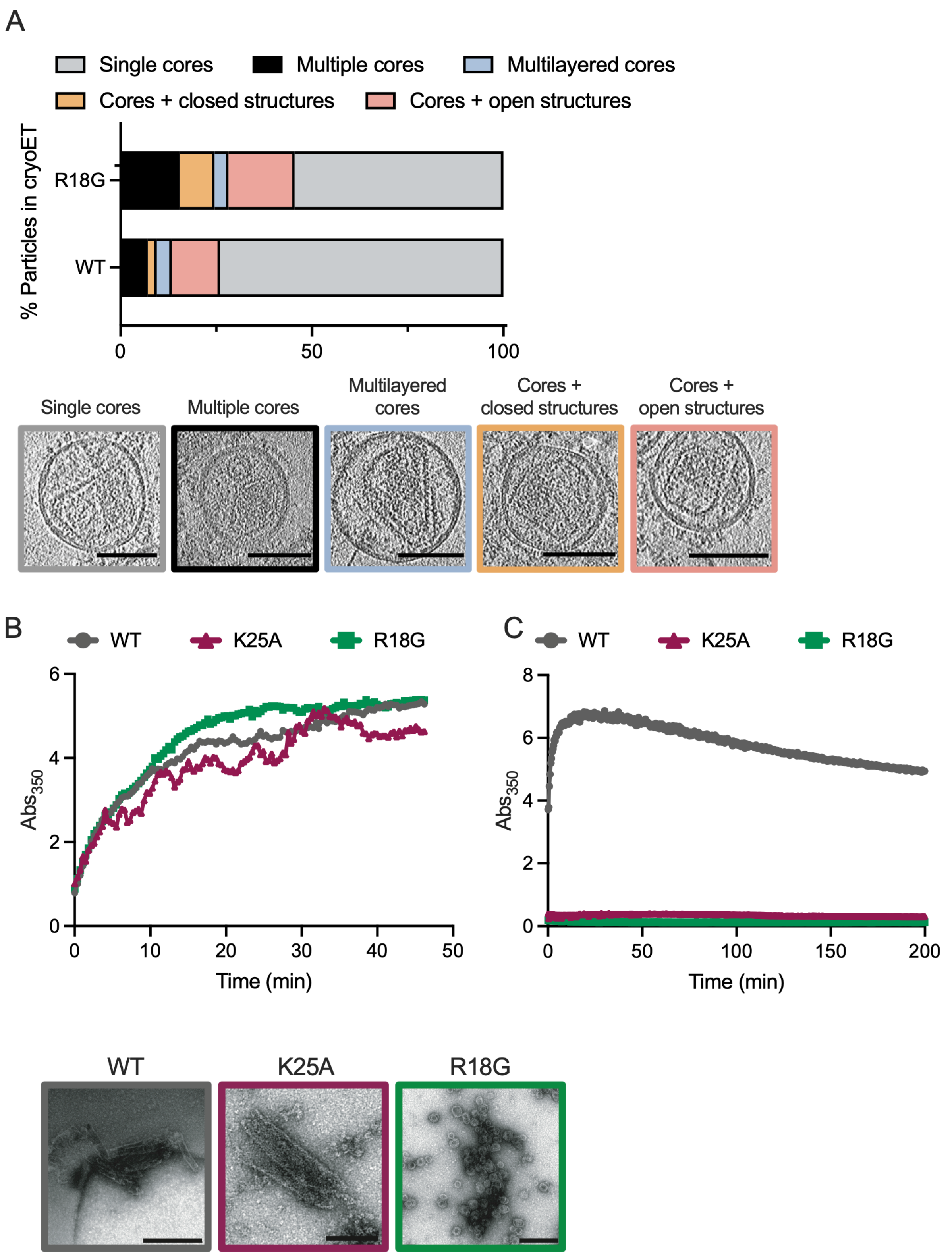

Supplementary Figure 1: Analysis of native and in vitro assembled HIV-1 virions. (A) Tomograms of virions from Figure 1B with mature lattices further subdivided into the following categories: multiple cores, single cores, cores with additional closed structure, cores with additional open structure and multilayered cores. Slices through example tomograms of the virions are shown together with the

proportion of each category. Scale bars, 100 nm. **(B&C)** In vitro assembly of 100 µM CA (WT, K25A and R18G) in 2.5 M NaCl (B) or 1.25 mM IP6 (C), measuring absorbance of the reaction over time at 350 nm. A representative (N=1) of three independent experiments is shown. Negative stain EM images of assembly reactions from (B) are also shown. Scalebar: 200 nm. Representative images from 3 independent experiments.

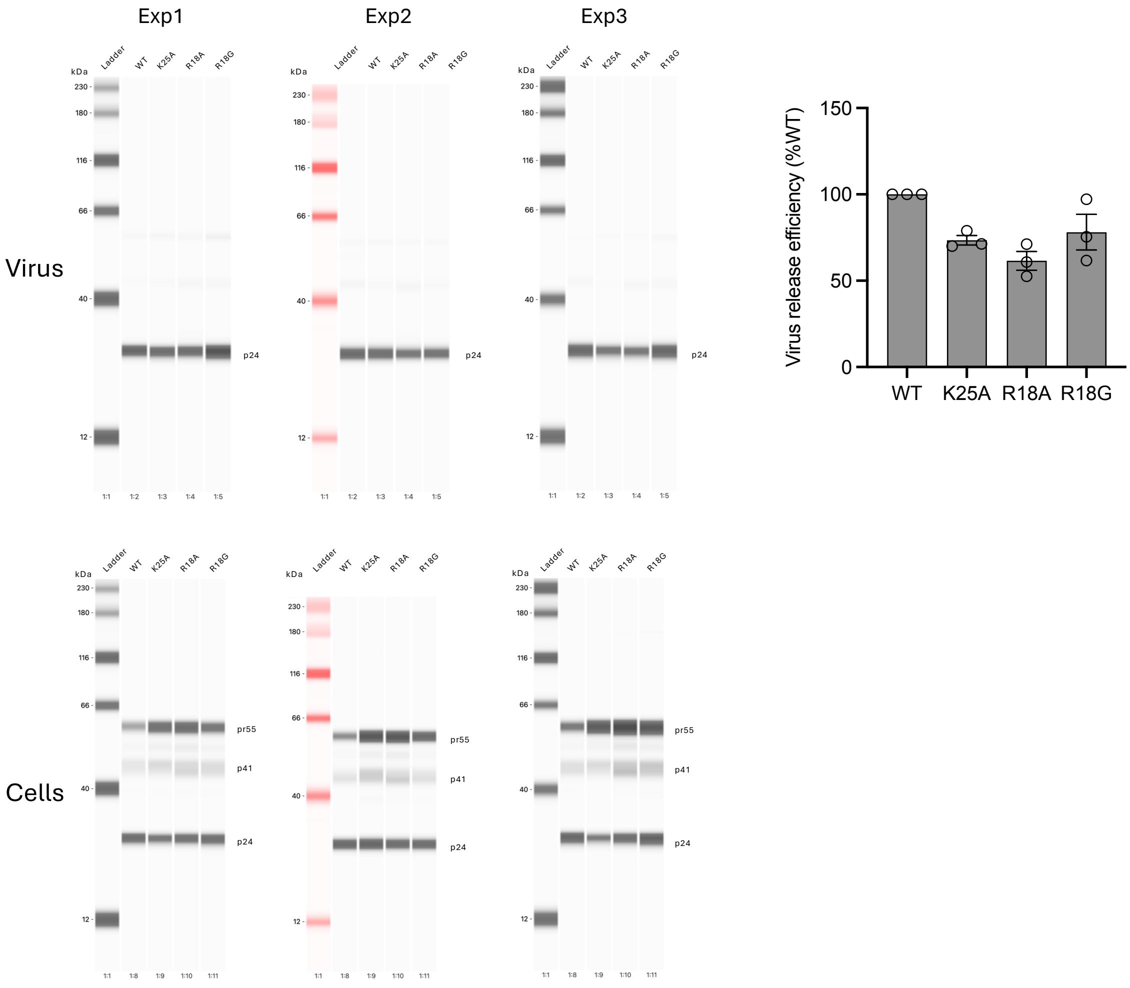

Supplementary Figure 2: Viral production of WT, K25A, R18A and R18G HIV-1. CA and Gag protein levels in virions and virus producing cells from three independent experiments. Virus release efficiency was calculated on the basis of the level of p24 in purified virions quantified as a percentage of p24 and Pr55 expression in producer cells and p24 expression in purified virions. Data are normalized to give the efficiency relative to WT virus. Error bars depict the SEM from the three independent experiments (N=3).

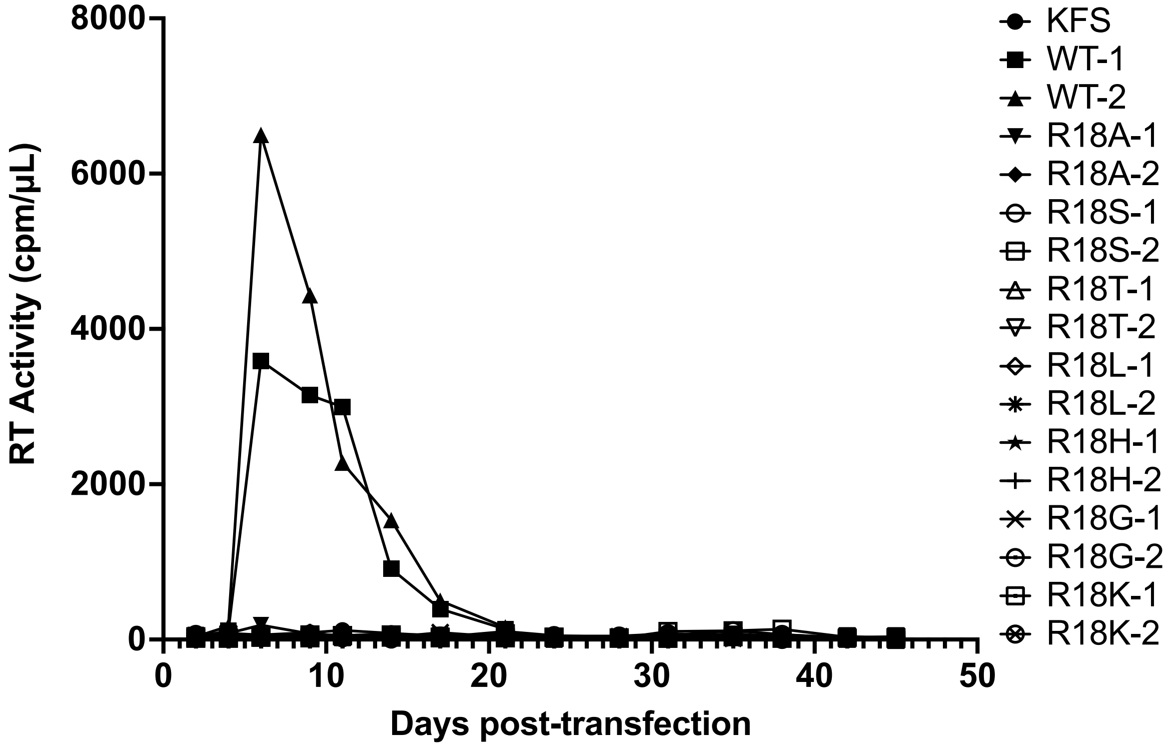

Supplementary Figure 3: Replication of R18 mutants. MT4 cells were transfected with infectious molecular clones (NL4-3) harboring a selection of R18 mutations in duplicate. Replication kinetics were assessed by quantifying supernatant RT activity. The molecular clone pNL4-3 KFS, which lacks a functional envelope (Env) gene, was included as a non-replicating control.

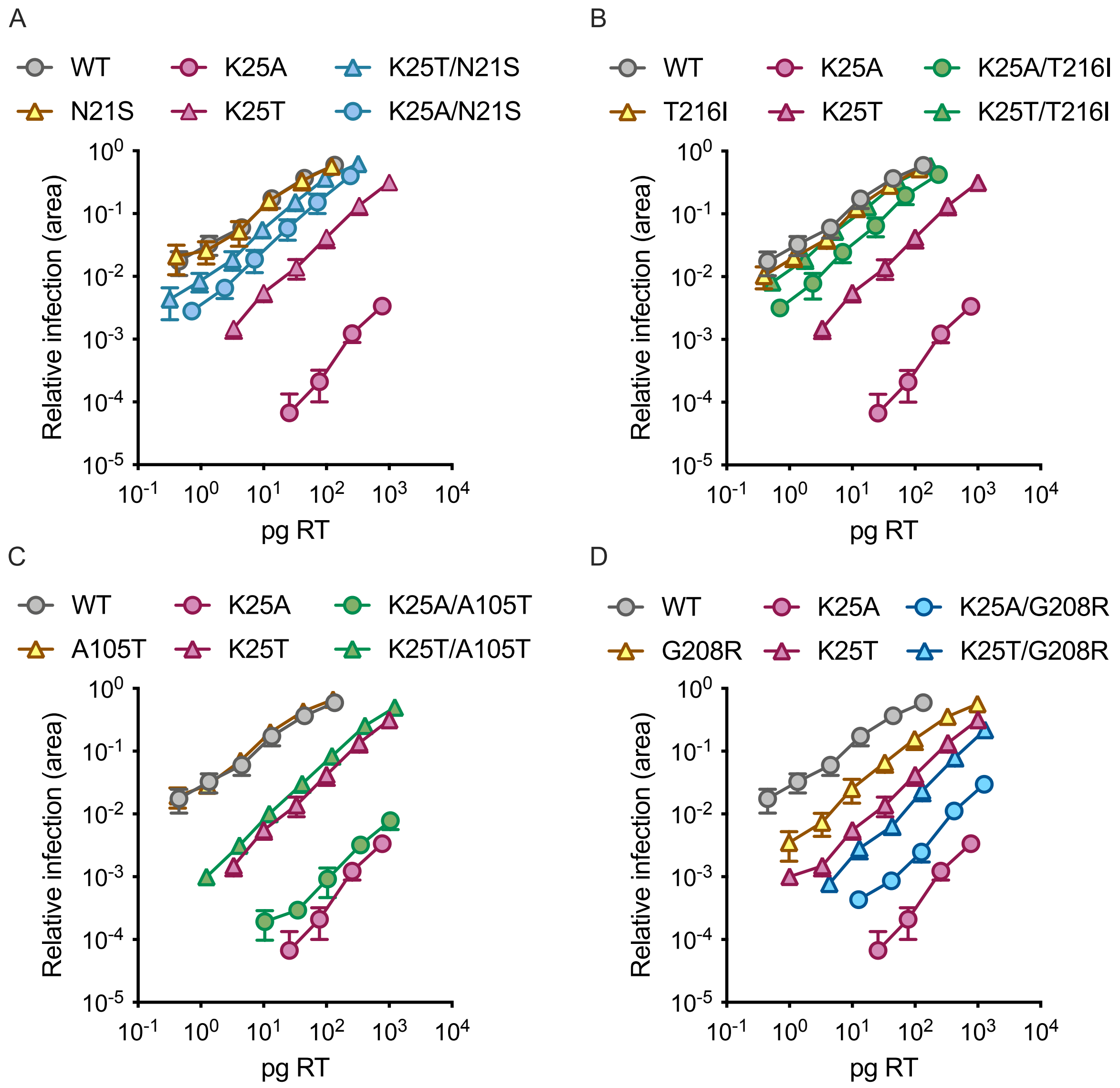

Supplementary Figure 4: Titration of K25A mutant viruses. (A-D) Single-round infection of the indicated viruses as measured by the proportion of infected cells (area of monolayer); N21S (A), T216I (B), A105T (C) and G208R (D) variants. Error bars depict mean CPM ± s.e.m. from three independent experiments (N=3).

Initial Transfection (MT4)	Re-passage 1	CA mutations	Re-passage 2	CA mutations
K25A (AAA --> GCG)	MT4	A25T		
	C8166	A25K	C8166	A25K
			SupT1	
	SupT1	No replication		No replication
K25A (AAA --> GCG)	MT4	T216I		
	C8166	T216I, G225S	C8166	T107I, T216I, G225S
			SupT1	T216I, G225S
	SupT1	T216I	SupT1	T216I
K25A (AAA --> GCG)	MT4	A25T		
	C8166	A25K	C8166	A25K
			SupT1	
	SupT1	A25K	SupT1	
K25A (AAA --> GCT)	MT4	A25T		
	C8166	A25T, N21S	C8166	A25T, N21S
			SupT1	A25T, N21S
	SupT1	No replication		
K25A (AAA --> GCT)	MT4	A25T		
	C8166	A25T, A105T, G208R, T216I	C8166	A25T, A105T, G208R, T216I
			SupT1	A25T, A105T, G208R, T216I
	SupT1	A25T, T216I	SupT1	A25T, T216I
K25A (AAA --> GCT)	MT4	A25T		
	C8166	No replication		
	SupT1	No replication		

Supplementary Table 1: CA mutations arising upon passage of K25A HIV-1 in three T cell lines. Sanger sequencing of genomic DNA isolated from infected cells collected from replicate propagation experiments (representative replication curves from Figure 2B and C) confirmed the acquisition of second-site mutations in CA upon passage of either K25A$_{GCG}$ or K25A$_{GCT}$ NL4-3 virus. Cell lines utilized for each viral passage and the observed CA mutations are listed. For re-passage 1, virus was harvested from the peak of replication in the initial MT4 transfection and used to infect the indicated cell lines. For re-passage 2, virus was harvested from the peak of replication in re-passage 1 and used to infect the indicated cell lines.
